# The Effect of Welfare State Policy Spending on the Equalization of Socioeconomic Status Disparities in Mental Health

**DOI:** 10.1177/00221465231166334

**Published:** 2023-04-25

**Authors:** Matthew Parbst, Blair Wheaton

**Affiliations:** 1 University of Toronto, Toronto, ON, Canada

**Keywords:** depression, social investment, social protection, socioeconomic status, welfare state

## Abstract

This article examines whether and how the relationship between socioeconomic status (SES) and depression is modified by welfare state spending using the 2006, 2012, and 2014 survey rounds of the European Social Survey (ESS) merged with macroeconomic data from the World Bank, Eurostat, and SOCX database (N = 87,466). Welfare state spending effort divided between social investment and social protection spending modifies the classic inverse relationship between SES and depression. Distinguishing policy areas in both social investment and social protection spending demonstrates that policy programs devoted to education, early childhood education and care, active labor market policies, old age care, and incapacity account for differences in the effect of SES across countries. Our analysis finds that social investment policies better explain cross-national differences in the effect of SES on depression, implying policies focused earlier in the life course matter more for understanding social disparities in the mental health of populations.

The inverse relationship between socioeconomic status (SES) and mental health is a cornerstone of the general argument linking social inequality to well-being (Adler et al. 1994; Aneshensel 2009; Pearlin 1989; Thoits 2010). The SES–mental health association is also one of the most consistent in social science (Thoits 2010), spanning over a century of research (Dohrenwend and Dohrenwend 1969; Faris and Dunham 1939; Hollingshead and Redlich 1958; Jarvis 1855; Srole et al. 1962) and continuing to the present day (Miech and Shanahan 2000; Warren 2009). This consistency within countries, predominantly estimated in the North American context, supports a social causation interpretation, net of social selection (Warren 2009; Wheaton 1978), indicating SES as a primary social determinant of mental health. However, cross-national comparisons across countries demonstrate that the strength of the association between SES indicators, such as education, occupation, and income, and mental health outcomes differs (Rai et al. 2013), implying contextual social determinants may be driving differences in the effect of SES between countries while also demonstrating the generality of the existence of this association across very different national contexts (Präg, Mills, and Wittek 2016).

At the same time, attention has also focused on the role of the institutional arrangements of welfare state policies as generalized social determinants of health, often due to their effects on SES (Beckfield et al. 2015; Ólafsdóttir and Beckfield 2020; Reynolds 2021). To assess the effects of policy, we utilize spending effort as an operational indicator of policy differences, recognizing that policies may exist with varying levels of funding and that the hypothetical presence of a policy without supportive spending is likely to have little to no effect (Castles 2008). Recent reviews demonstrate that the spending effort in general areas of social policy is associated with SES differences in mental health, where increases in spending generally reduce SES differences both between (McAllister et al. 2018) and within countries (Simpson et al. 2021). Between-country differences highlight the importance of contextual differences in policy environments for SES inequalities in mental health, averaged over time, whereas within-country analysis demonstrate changes in either the direction or magnitude of specific policy effects over a given period. Between-country studies find greater levels of active labor market (Niedzwiedz et al. 2016) and sickness benefit spending (van der Wel et al. 2015) reduce educational differences in mental health. Within-country analyses demonstrate that SES inequalities are impacted by the direction of spending effort so that increases reduce and reductions increase inequalities across family policies (Lebihan and Mao Takongmo 2018; Milligan and Stabile 2011), social assistance (Herbst 2013), and old age security (Carrino, Glaser, and Avendano 2020).

Our approach incorporates both between- and within-country effects of policy by using cross-level interactions to understand if cumulative historical realities or short-term changes through time in policy investment have distinct and/or differential impacts on the association between SES and mental health at the individual level (Bell and Jones 2015). Each taps a distinct realm of the role of state policy. Between-country differences, partitioned from within-country differences over time, reflect overall averaged differences across countries that persist through time. Between-country differences allow for the identification of cumulative impacts more straightforwardly. For example, the cumulative effects of relatively stable levels of investment in childcare could affect both the consequences of having children and the expectation of support if you do not but foresee this possibility. This scenario suggests that policy investments can have indirect effects on decision-making and experience at the individual level, possibly encouraging entry into the labor force. Providing access to labor force entry can in turn reduce the worst consequences of low SES for mental health. Within-country differences reflect the effects of changes in policy within countries over time. In this case, recent changes in access options may be as important as the level of support, following the hypothesis that changes in resources can affect well-being as much as or even more than the level of resources (Davis 1984). In our approach, both level of and changes in spending are separated and captured as distinct possibilities. An important issue this allows for is the case where policy patterns between and within countries trend in opposite directions, resulting in conflated and counteracting forces that could mask the effects of each. This could happen, for example, when countries with historically high levels of spending effort in a particular social policy—resulting in a weaker association between SES and mental health—begin spending less over time, thus reactivating the link between SES and mental health.

We argue that distinguishing cumulative historical levels of spending from within-country trends provides a more holistic and thus complete theoretical understanding of how policy processes may impact the relationship between individual social status (or position) and mental health. Our approach also implies that the impact of policy on mental health is not ahistorical. It is important to resolve whether the current state of linkages between social statuses and mental health depend on current funding or the historical carry-forward of the norms set in place by average levels of funding through time.

As such, we seek to add to the institutional perspective on the role of social policy in the association between SES and mental health by asking: What policies are capable of weakening that association—and thus of reducing the mental health risk for lower SES groups—both historically between countries and via trends in spending over time within countries? In general, we specify these effects as different cross-level interactions in a three-level multilevel model using an approach suggested by Bell and Jones (2015) and Giesselmann and Schmidt-Catran (2019).

In answering this question, we focus on both the social protection and social investment policy paradigms and their impacts on SES inequalities. First, social protection policies seek to mitigate the unforeseen and adverse life circumstances accompanying disadvantaged statuses by promoting a minimum standard of living (Titmuss 1974; Wilensky 1975). The main mechanism of social protection policies is the transfer of income to individuals or households through either the traditional focal policies of the paradigm, such as pensions, sickness, and unemployment benefits (Esping-Andersen 1990), or the expanded policies of transfers to families and welfare services, such as health care and housing (Castles 2008; Esping-Andersen and Myles 2009). Second, the social investment paradigm focuses on policies that promote human capital formation and increased household labor market participation (Esping-Andersen et al. 2002; Hemerijck 2017). Focal policies include early education and care programs (ECEC), general education, postsecondary education and vocational training, active labor market policies (ALMPs), and old age care (Kvist 2015). Both perspectives ultimately focus on methods by which welfare states reduce inequality and poverty (Brady 2009; Plavgo and Hemerijck 2021) but highlight different pathways to achieve these outcomes. We argue that the differences in the mechanisms involved in these pathways may have differential consequences for the link between SES and mental health. Social investment policies focus on individual-level prevention of pathways leading to inequality and poverty, such as poor education and labor market attachment. Social protection policies reflect reactive policies and are more responsive to immediate needs, for example, by transferring income or the provision of services when individuals cannot meet their needs from the labor market.

We focus on depression as the mental health outcome. Even though we necessarily focus on a single measure, variations in depression in a population remain a central marker of differences in the mental health of populations overall given its prevalence, its seriousness and chronicity as a problem, and its overlap with associated mental health indicators, such as anxiety (Kessler et al. 1995, 2005; Mirowsky and Ross 2003). By studying depression, we also focus on the state’s role in the alleviation of suffering rather than the maximization of well-being, which may be more volatile in general populations (Diener and Diener 1996) and also depends more heavily on interpersonal rather than macro-social inputs (Diener et al. 1999; Veenhoven 2000).

We assess each component of SES on mental health both across general policy processes of the welfare state and by disaggregating each of the processes to determine first, which components of SES are affected most and second, which kinds of specific policies have the most significant impact. As such, we argue our study makes three contributions to the research tradition linking SES to mental health. First, it assesses whether the relationship between SES and depression varies significantly across and within 21 modern welfare states. Second, this study specifies and tests some of the institutional policy mechanisms (indicated by social investment vs. social protection spending effort) utilizing cross-level interactions that allow for the detection of differential policy effects on the relationship between SES and depression and that are beyond what can be considered in a main effects analysis of SES differences (Bell and Jones 2015). Third, we disaggregate specific policies in these policy paradigms to specify better which areas of the welfare state matter most in reducing SES-based disparities in mental health. Overall, our results suggest that greater social policy spending effort provides “cumulative” mental health advantages (Thoits 2010) at the individual level.

## Background

### SES and Mental Health at the Individual Level

Education is argued to be the primary causal force linking SES to mental health across the life course (Mirowsky and Ross 2007). A central role of education in the production of mental health is the socialization of beliefs and skills that together result in higher levels of important personal coping resources, such as cognitive flexibility or a sense of control over life outcomes (Ross and Mirowsky 2010; Wheaton 1983). Those with high education tend to have more complex jobs, resulting in more intellectual flexibility (Kohn and Schooler 1982). Education also has indirect effects through its effect on economic resources, such as workplace authority and income, that help maintain better mental health (Kessler 1982; Turner, Wheaton, and Lloyd 1995). However, the mental health benefits to education are not limitless. There are potentially diminishing returns to education on mental health among the highly educated, who receive low economic returns relative to their educational attainment (Bracke, Van De Straat, and Missinne 2014).

Whereas increasing education predominantly places individuals in advantageous life circumstances beneficial for mental health (Miech and Shanahan 2000; Ross and Mirowsky 2010), occupation (Kohn et al. 1990) and income (Lynch, Kaplan, and Shema 1997) also may have independent effects on mental health. Occupational status, for example, reflects structural job conditions that have both direct and indirect effects on mental health (Kohn and Schooler 1973; Link, Lennon, and Dohrenwend 1993), including the mental health advantages of more complex, more creative, and more meaningful work also involving greater control over the terms of work relative to lower status jobs that are routine and manual in nature (Kohn et al. 1990). Higher incomes decrease depression by reducing levels of financial strain and economic hardship (Mirowsky and Ross 2003) but more broadly, by creating choice and thus fostering a sense of mastery. Finally, SES in general structures the availability of social resources individuals may draw on (Thoits 1995), particularly through the effect of income on access to social capital (Song 2011).

### Cross-National Welfare State Impacts on SES and Generalized Health

Studies on the impact of welfare states on SES have examined self-reported health (Bambra et al. 2009; Bambra, Netuveli, and Eikemo 2010; Dahl and van der Wel 2013; Eikemo et al. 2008), the World Health Organization mental well-being index (van der Wel et al. 2015), and depression (Hansen, Slagsvold, and Veenstra 2017; Niedzwiedz et al. 2016). A majority of the research in this area utilizes Esping-Andersen’s (1990) three regime typology to test differences in the welfare state’s impact of SES on health (Bergqvist, Yngwe, and Lundberg 2013), although studies focusing on social protection spending find greater spending reduces educational differences in self-rated health (Dahl and van der Wel 2013) and mental well-being (van der Wel et al. 2015).

Two cross-national studies focus on the relationship between education and depression. Niedzwiedz et al. (2016) find a moderating effect of active labor market spending on the association between levels of education and depression. However, evidence for this conclusion can be questioned because they do not consider the random (unmeasured) effects of countries or time due to the pooling of waves in their sample, thus potentially conflating between- and within-country processes. Finally, Hansen et al. (2017) utilized separate ordinary least sqaures regressions to calculate the odds of the risk of depression for seniors (age 60–80) for 10 countries. Although not testing any policies directly, they observe smaller differences in the effect of education on depression in socially democratic welfare states and the largest within post-Soviet states.

### Research Questions

We ask two main research questions and consider the relative importance of social investment and social protection in each of them. Our first research question addresses considerations of the average differences between countries and their effects on mental health: Are enduring differences in the spending levels between countries associated with a weakening of the association between SES and mental health?

This question emphasizes the importance of considering average policy climates across time. Policy levels are relatively stable over short periods due to “path dependency” logic where removal or reduction of benefits is contested (Pierson 2000). We expect to find enduring differences between countries that continuously spend higher versus lower amounts on social investment and social protection spending. We argue that this expectation reflects a “cumulative advantage” for mental health, especially among individuals in societies where continuous investment at high levels promotes overall relative structural advantages that individuals may benefit from, especially for those with low status. Whereas changes over time in a country test the impact of the direction and magnitude of social policy effects, the average context between countries assesses differences due to generalized differences in the levels of social policy that persist through time (Thoits 2010).

Our second question focuses on the impact of the direction and magnitude of changes in social policy over time: Do changes in social investment and social protection policies help account for variation in the inverse association between SES and mental health in countries over time? In assessing both questions, we assess which of our two types of policies are most relevant to the weakening of the link between SES and mental health and the specific policies that may be doing so. Our data cover a relatively short historical period (2006–2014). As a result, this may hamper our ability to detect the full impact of changing policies, especially because path dependency logic suggests that change is incremental and takes time. Nevertheless, we contend that explicitly separating between- and within-country trends is important both conceptually and empirically (Bell, Fairbrother, and Jones 2019). This question parallels the classic status versus change issue in the literature on general well-being: Is it level of income or changes in income that are most relevant to well-being in general (Davis 1984)?

Both questions we address imply that some combination of social investment and social protection spending will modify the SES mental health association. We assess this by examining the moderating impact of social investment and social protection spending and their component policies for each component of SES on depression.

## Data and Methods

Individual data were taken from the 2006, 2012, and 2014 survey rounds of the European Social Survey (ESS) of representative samples of adults (restricted to 18 and above). Rounds had to be specifically chosen based on the availability of depression measures. Rounds varied in sample size from 35,210 to 42,351, and typical within-country samples per year varied from 949 to 2,925 and were combined for a total pooled sample of 113,078. Included in this study were 21 countries: Austria, Belgium, Czech Republic, Denmark, Estonia, Finland, France, Germany, Ireland, Israel, Lithuania, Netherlands, Norway, Poland, Portugal, Slovakia, Slovenia, Spain, Sweden, Switzerland, and the United Kingdom. Countries excluded from this analysis were Bulgaria, Cyprus, Hungary, Iceland, Ukraine, and the Russian Federation, either due to the unavailability or quality of welfare state data or inclusion in the ongoing ESS at only one point in time. Using listwise deletion, we obtained a total analytic sample of 83,091, with attrition occurring mainly from occupation (6,588) and income measures (16,496).

### Dependent Variable

*Depression* was an averaged index of eight survey items taken from the widely used Center for Epidemiologic Studies Depression Scale (CES-D; Van de Velde et al. 2010). The eight CES-D scale items used here load consistently on a single latent construct and behave similarly across countries and demographic groups (Van de Velde et al. 2010). We adjusted averaged item scores to range from 0, or no weekly symptoms of depression, to 24, which represents reporting the most severe response on every survey item.^1^ Each CES-D item question asked respondents to report on the last week only. Question items included were: felt depressed, felt everything you did was an effort, your sleep was restless, you were happy, you felt lonely, you enjoyed life, you felt sad, and you could not get going. Depression had a Cronbach’s alpha of .84.

### SES

*Education* was measured in years ranging from 0 to 25.^2^ We included a squared education term to account for diminishing returns to education (Bracke et al. 2014). *Household occupational status* translated ISCO-88 occupational codes in the 2006 survey wave and ISCO-08 occupational codes in 2012 and 2014 surveys into the International Socio-Economic Index of Occupational Status (ISEI) scale (Ganzeboom and Treiman 1996) utilizing the updated ISEI-08 coding (Ganzeboom 2010). The ISEI index was applied to the respondent’s reported occupation and, if indicated, their partner’s occupation returning the highest score ranging from 1.10 to 8.90. Finally, *household income* was calculated in deciles for both survey years and ranges from 1 to 10.^3^

### State-Level Moderators

Following Kuitto (2016), *social investment* was a measure combining four areas of welfare state spending reported as a percentage of total gross domestic product (GDP): education, ECEC, ALMP, and old age care. Education spending was extracted from the World Bank (2021).^4^ ECEC, ALMP, and old age care were extracted from the SOCX Organization for Economic Cooperation and Development (OECD) spending database (OECD 2021).^5^ Also following Kuitto (2016), we measured *social protection spending* as a percentage of GDP of total cash transfers spending on incapacity, old age, survivors, unemployment, and other social exclusion spending, taken from the SOCX database (OECD 2021).^6^ However, we also included service spending on health care and housing in this category, also taken from the SOCX database, due to its effects on poverty and inequality and thus effects on a minimum standard of living (Esping-Andersen and Myles 2009). We present the disaggregation of each policy paradigm in Table 1.

**Table 1.** Social Investment and Social Protection Policy Paradigm Overview.

Policy Paradigm	Disaggregated Policies
Social investment	Early childhood education and care (ECEC) Education Active labor market policies Old age care
Social protection	Family transfers Unemployment transfers Old age transfers Survivors transfers Incapacity transfers Health Housing

### Controls

Controls in this analysis included confounders not on the causal pathway between individual SES and depression, such as age and gender (Mirowsky and Ross 2003) and father’s educational status, to incorporate the intergenerational transmission of SES. At the country level, we controlled for economic development as measured by GDP per capita based on purchasing power parity (divided by 1,000) in constant 2011 international dollars taken from the World Bank (2021) and income inequality, as measured by the GINI coefficient for household market income, ranging from 0 to 1 where 1 equals complete inequality (Solt 2019).^7^ Both country measures impact psychological well-being (Wilkinson and Pickett 2010). Finally, we estimated the effects of each category of spending, controlling for the other category in the same model. Thus, for example, if we estimated the effect of social investment in general or a component of social investment (education spending), this was in the presence of the associated effects of social protection.

Finally, we considered each ESS round as a control allowing for the observation of secular changes in our dependent variable across time.

### Analysis

We employed three-level hierarchical linear models (HLMs) to answer our research questions, incorporating both between-country averaged differences over time and within-country trends. In these models, the individual was Level 1, the country-year was Level 2, and the country was Level 3 (Giesselmann and Schmidt-Catran 2019). Our analytic strategy addressed core issues in using multiple cross-sectional, cross-national studies to estimate differences between countries *and* over time within countries using pooled time-series cross-sectional data structures and small number of countries (Schmidt-Catran, Fairbrother, and Andreß 2019). We followed the approach of Giesselmann and Schmidt-Catran (2019) to estimate our cross-level interactions between spending and SES but extended their model to include a random coefficient for SES at both the country (Level 3) and country-year levels (Level 2) utilizing a within–between coding structure (Bell and Jones 2015). We did so because first, it is consequential for estimates of Level 3 variables in the model (Heisig, Schaeffer, and Giesecke 2017) and second, to account for unobserved differences in the effect of SES on depression both between and within countries (Bell et al. 2019). The use of the three-level model along with the between and within coding in HLM allowed for the simultaneous estimation of both effects.

Our HLM strategy also accounted for small cluster sizes (Level 3 N) by utilizing a Satterthwaite approximation following Elff et al. (2021) given our number of countries was 21. All models used ESS poststratification weight. We describe a full accounting for the model structure in Appendix A in the online version of the article.

In our models, we distinguished “between” from “within” effects. This distinction had a somewhat different meaning applied to state-level policy differences versus individual measures of SES. At the state level, the between measure reflected the average level of spending over time at the country level, while the within measure reflected changes in levels of spending in each country over time. At the individual level, the between measure of SES in effect controlled for the average level of SES over time in each country, while the within measure reflected the relative position of individuals on SES relative to that mean. Including the between effect of SES controlled for the compositional mean of SES across countries. Individual differences in SES were captured by the within measure—analogous to a group-mean centered approach at the individual level, but with the group mean included in the model (Hox 2010).

### Robustness Checks

Following Schmidt-Catran et al. (2019) and Van der Meer, Te Grotenhuis, and Pelzer (2010), we conducted an outlier analysis and report the Cook’s D in Appendix B in the online version of the article. All countries with a Cook’s D value higher than 4/N were removed from analysis to examine their influence on model results, and any model modifications are noted in tables. Outlier model results are available by request.

## Results

### Descriptive Overview

Descriptive information in Table 2 highlights the general trends of the combined sample. Beginning with individual-level variables, depressive symptoms remain constant through Round 3 and Round 6 at 5.61 symptoms per week and decrease to 5.25 in Round 7 with relatively consistent variation across waves. Turning to controls, over half of the sample is female in all three waves. From the 2006 to 2014 waves, there is an increasing percentage of individuals whose father’s education is above secondary, ranging from 15% in Round 3 to 19% in Round 7. The average age increased slightly, from nearly 49 years to around 51 years. Finally, SES trends show both education and occupation slightly increasing over time while income levels marginally decreased on average.

**Table 2.** Descriptive Overview of European Social Survey Rounds 3, 6, and 7.

	Round 3 (*N* = 24,702)		Round 6 (*N* = 29,950)		Round 7 (*N* = 28,439)	
	Mean or % (SD)	Range	Mean or % (SD)	Range	Mean or % (SD)	Range
**Dependent variable**						
Depression	5.61 (4.01)	0–24	5.60 (4.12)	0–24	5.24 (3.96)	0–24
**Individual-level variables**						
*Controls*						
Female	52%		52%		52%	
Father’s level of education						
Above secondary	15%		19%		19%	
Age	48.82 (16.90)	18–97	5.44 (17.09)	18–102	5.83 (17.23)	18–114
*Focal independent variables*						
Education (years)	12.67 (4.12)	0–25	12.99 (3.98)	0–25	13.18 (3.93)	0–25
Household occupation	4.68 (2.18)	1.10–8.90	4.63 (2.20)	1.10–8.90	4.74 (2.22)	1.10–8.90
Household income (deciles)	5.61 (2.45)	1–10	5.31 (2.77)	1–10	5.35 (2.75)	1–10
**Country-level variables**						
*Controls*						
Gross domestic product (thousands)	39.85 (10.77)	18.27–63.85	37.57 (10.23)	23.22–63.00	40 (9.43)	25.31–63.42
Gini	.47 (.04)	.39–.52	.48 (.04)	.37–.52	.47 (.04)	.37–.52
*Focal moderators*						
Social protection	18.47 (3.92)	11.91–25.45	19.73 (4.43)	13.30–28.92	2.06 (4.41)	13.23–28.97
Family cash	1.03 (.48)	.32–2.08	.98 (.61)	.20–2.43	.98 (.56)	.18–2.10
Unemployment	1.01 (.76)	0–3.16	1.09 (.98)	0–3.24	1.05 (.88)	0–3.24
Old age cash	6.67 (2.13)	2.40–10.33	7.34 (2.09)	4.20–12.20	7.78 (2.37)	4.12–12.25
Health	5.48 (1.44)	2.45–7.92	5.96 (1.54)	2.95–8.72	6.13 (1.60)	2.91–8.81
Survivors cash	1.04 (.76)	0–2.16	.91 (.76)	.01–2.34	.96 (.75)	.01–2.34
Incapacity cash	2.16 (1.04)	1.22–3.42	2.01 (.49)	1.53–3.31	2.00 (.54)	1.28–3.29
Housing	.43 (.44)	0–1.78	.54 (.73)	0–3.32	.41 (.39)	0–1.54
Social investment	7.29 (1.95)	5.03–12.31	7.57 (2.31)	4.89–12.90	7.67 (2.31)	5.01–12.93
Early education and care programs	.64 (.35)	.25–1.33	.75 (.37)	.32–1.58	.78 (.36)	.38–1.64
Education	5.37 (.90)	3.72–7.73	5.62 (1.11)	3.91–7.66	5.63 (1.12)	3.99–7.67
Active labor market policies	.69 (.33)	.07–1.46	.65 (.42)	.14–1.93	.69 (.43)	.13–2.03
Old age care	.56 (.65)	0–2.13	.54 (.65)	0–2.16	.57 (.66)	0–2.18

Country-level controls are mostly stable across waves except for GDP per capita. GDP per capita declined in Round 6 and then increased beyond Round 3 levels in Round 7. Gini remained relatively constant across waves.

Social protection and investment spending increased across this time, and a nontrivial amount of spending differences exists across areas. Comparatively, states spend more on social protection spending on average. Social protection spending also generally has the larger average increase across waves, with a proportionate increase of about 2% of GDP. In social protection spending, the two largest areas of spending are old age cash benefits and health. These two areas have driven the overall increase in social protection because all other programs remain relatively constant. Social investment only marginally increased, with less than a 0.5% increase over this period due to marginal increases in education and ECEC. All within-country trends are in Appendix C in the online version of the article.

### Estimating the Role of State-Level Policies in the SES–Depression Relationship

Table 3 examines the relationship between the three indicators of SES and depression. Random coefficient models are presented to reflect the possibility that the effect of SES varies across countries, based on unspecified country-level differences. The test of the random variation of coefficients for SES is a logical first step that signals the possibility, or not, of specific cross-level interactions. We also include a country-year random slope that acknowledges within-country trends.

**Table 3.** Random Coefficient Models’ Fixed and Random Effects for Socioeconomic Status Predicting Depression (*N* = 83,091).

	(1) Education	(2) Household Occupation	(3) Household Income
**Fixed components**			
Intercept	8.248 (5.614)	9.116 (5.593)	8.149 (5.806)
*Controls*			
Round 6	−.255** (.079)	−.297*** (.070)	−.242* (.102)
Round 7	−.424*** (.081)	−.488*** (.071)	−.414** (.104)
Female_W_	.837*** (.026)	.837*** (.026)	.713*** (.026)
Female_B_	4.562 (5.985)	8.241 (6.005)	10.653 (6.825)
Father’s education			
Above secondary_W_	.027 (.037)	.143*** (.037)	.186** (.037)
Above secondary_B_	1.729 (2.514)	5.264 (2.739)	5.396 (3.234)
Age_W_	.008*** (.001)	.013*** (.001)	.008*** (.001)
Age_B_	.013 (.098)	−.048 (.096)	−.081 (.117)
*Focal associations*			
Education_W_	−.436*** (.062)	−.289*** (.016)	−.247*** (.015)
Education_B_	−.716 (.510)	.485 (.498)	.368 (.526)
Education_W_^2^	.011*** (.002)	.009*** (.001)	.008*** (.001)
Education_B_^2^	.019 (.025)	−.024 (.024)	−.018 (.025)
Occupation_W_		−.243*** (.019)	−.123** (.008)
Occupation_B_		−1.765** (.538)	−1.481* (.565)
Income_W_			−.288*** (.020)
Income_B_			.067 (.349)
**Random components**			
*Between country*			
Intercept	.877* (.430)	.311* (.134)	.386* (.202)
Education	.071** (.028)		
Education^2^	.000* (.000)		
Occupation		.006** (.002)	
Income			.006** (.003)
*Within country*			
Intercept	.051*** (.015)	.054*** (.016)	.087*** (.025)
Education	.001* (.000)		
Occupation		.002* (.001)	
Income			.004** (.001)

*Note*: All within-country effects are denoted by _W_, and all between-country effects are denoted by _B_. Data are from Rounds 3, 6, and 7 of the European Social Survey.

* *p* < .05. **p < .01. ***p < .001 (two-tailed).

Models for the fixed effects of SES, including the random effects noted previously, are tested in Models 1 through 3—one each for each component of SES. In Models 1, 2, and 3, we build a model for the fixed effects of SES by considering the standard causal sequence for education, occupation, and income (Sewell, Hauser, and Featherman 1976). Thus, in the model testing the fixed and random effects of occupation, we control for education and include a random effect for occupation (Model 2), and in the model from income, we control for both education and occupation and include a random effect for income (Model 3). The fixed effects in these models include controls at the individual level for the survey round,^8^ gender, age, and father’s education.

Our focus in Model 1 is on the within and between effects of education and the squared term for the possibility of diminishing returns of education. The focus of our research questions is on the within measures at the individual level, while the between measures capture aggregate differences in average SES only but must be controlled. Table 3 shows that the within differences in education are negatively related to depression, while the between differences are not. This is consistent with expectation. State-level differences in SES are likely to be smaller than individual differences within countries. The squared education term at the individual level is also significant in the expected positive direction, indicating a diminishing return to high levels of education. This model also shows a significant random slope component of the effect of education between countries and within countries, signaling that this association does vary across countries, and this may signal important variation in the effect of SES due to state-level differences. Finally, the random effect for the squared education term is only significant at the between-country level.

Models 2 and 3 add occupational status and income progressively to the education model. The effect of occupational status before adding income is notably larger than in the final model—reflecting partial mediation by the effect of income—and again, the random coefficients in this model are significant, in this case, both between and within countries. Model 3 also indicates that there is a between effect of occupation. Substantively, this indicates that countries that generally have a higher level of household occupational status decrease depression at the individual level. Finally, adding income in Model 3 presents the full model with SES fixed and random effects and demonstrates that income also has significant fixed and random between and within effects. Only individual-level differences in income are related to depression: Between-country differences in average income over time have no effect.

All SES components in this final model have distinct negative associations with depression—no one component absorbs the effects of other components. This finding is important because analyses only using one or two components will underestimate the total effect of SES. Overall, Table 3 demonstrates a consistency with SES findings in the literature and the generality of its association with mental health within countries. At the same time, the significance of the accompanying random effects for all components of SES also suggests the possibility of cross-level interactions with state-level policy differences.

Table 4 presents models that include both individual-level and contextual effects and thus assess both of our research questions. Results are presented separately for within versus between components of both SES and spending, although again, our focus is on the within effects. Each component of SES includes two models: the first testing the effect of social investment (SI) and the second testing the effect of social protection (SP) while also controlling for the other form of spending.

**Table 4.** Between and Within Moderating Fixed Effects of Social Investment and Social Protection Spending on Socioeconomic Status and Depression (*N* = 83,091).

SES Component	Education		Household Occupation		Household Income	
Welfare State Policy	SI	SP	SI	SP	SI	SP
Intercept	9.133 (5.365)	8.990 (5.335)	12.242* (4.971)	12.291* (4.905)	12.244 (5.469)	12.067 (5.46)
**Individual level**						
*Focal associations*						
SES Component_w_	−1.035*** (.159)	−.641 (.300)	−.411*** (.053)	−.307** (.089)	−.497 (.052)	−.311** (.099)
SES component_b_	−.303 (.480)	−.298 (.477)	−1.206 (.576)	−1.201 (.568)	.428 (.350)	.407 (.349)
Education^2^_w_	.026*** (.005)	.015 (.009)	.009*** (.001)	.009*** (.001)	.008*** (.001)	.008*** (.001)
Education^2^_b_	.007 (.024)	.007 (.024)	−.000 (.022)	.001 (.021)	.007 (.025)	.006 (.025)
**Contextual level**						
*Controls*						
GDP per capita_w_	−.087 (.043)	−.097* (.045)	−.091* (.042)	−.090* (.041)	.020 (.065)	.006 (.065)
GDP per capita_b_	−.067** (.016)	−.068** (.016)	−.053** (.015)	−.053** (.015)	−.051* (.017)	−.051* (.017)
GINI_w_	5.092 (3.121)	4.868 (3.140)	3.716 (3.025)	3.332 (2.996)	3.934 (4.634)	3.623 (4.733)
GINI_b_	1.561 (3.330)	1.551 (3.311)	−1.155 (3.334)	−1.135 (3.290)	.259 (3.789)	.118 (3.783)
*Focal moderators*						
Social investment_w_	−1.676 (2.182)	−.038 (.111)	−.304 (1.559)	.004 (.098)	−1.388 (1.027)	−.120 (.153)
Social investment_b_	−.107 (.072)	−.118 (.070)	−.084 (.067)	−.104 (.065)	−.138 (.076)	−.119 (.076)
Social protection_w_	−.029 (.040)	−.248 (.294)	−.040 (.044)	.129 (.274)	.015 (.068)	−.041 (.257)
Social protection_b_	.022 (.037)	.028 (.037)	.004 (.033)	.007 (.033)	.012 (.037)	.011 (.037)
Interactions						
SES_b_ × Policy_w_	.128 (.166)	.017 (.022)	.068 (.323)	−.036 (.061)	.220 (.180)	.002 (.050)
SES_w_ × Policy_b_	.082** (.021)	.011 (.015)	.023** (.007)	.003 (.005)	.029*** (.007)	.001 (.005)
Ed^2^_w_ × Policy_b_	−.002* (.001)	.000 (.000)				
SES_w_ × Policy_w_	−.007 (.012)	−.002 (.004)	−.023 (.019)	−.007 (.006)	.025 (.023)	.001 (.007)

*Note*: All within-country effects are denoted by _w_, and all between-country effects are denoted by _b_. Data are from Rounds 3, 6, and 7 of the European Social Survey. SI = social investment; SP = social protection; SES = socioeconomic status; GDP = gross domestic product; Ed = education.

* *p* < .05. **p < .01. ***p < .001 (two-tailed).

Results include controls for the contextual effects of GDP and Gini. Except for the household income models for SI and SP spending and the education model for SI, the within effect of GDP per capita is negative and significant, indicating that increasing economic development levels across time reduce depression. There are also negative between effects for GDP per capita across all models. This indicates that countries with historically higher GDP per capita also tend to have lower levels of depression. However, counter to expectations, the effect of income inequality—as measured by Gini—is not significant at either the between or within level of analysis.

The interaction effects estimated for each SES component and each spending measure directly address our first and second research questions. We consider first the interaction between differences in SES and spending within countries, primarily to address potential unobserved confounding (Giesselmann and Schmidt-Catran 2019). None of these interactions are significant, as expected. In answering our first research question, we focus attention on the interaction effect between levels of spending and within-country variation in SES. All significant interactions in these results involve SI spending. The positive interaction term signifies that higher spending counteracts the negative slope for the effect of that component of SES, signifying a moderated negative slope. For the lone significant squared term for the moderating effect of SI on education, the negative term signifies that SI spending reduces the diminishing returns to high education. The most consistent result here is the interaction between individual differences in SES and overall social investment differences between countries over time. This finding suggests that a general climate of social investment does reduce depression differences between high and low SES at the individual level. In results not displayed, we note that all SI models explain away the negative depression trend across waves, suggesting it to be an important mechanism for societal well-being.

Regarding our second question, we fail to detect any within-country trends in policy that mitigate the effects of SES across the observed period in these data. We noted earlier the possible problems of detecting these sources of influence over delimited time frames.

**Figure 1.**
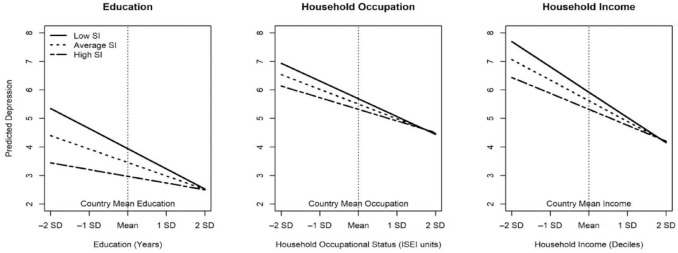
Between-Country Moderating Effect of Social Investment Spending on Education, Occupation, and Income on Depression. *Note*: Data are from Rounds 3, 6, and 7 of the European Social Survey.

We display the moderating between-country effect of social investment spending on the impact of SES in Figure 1. This figure displays predicted values of depression by levels of education, occupation, and household income, respectively, at three levels of SI spending—low, average, and high, where “low” and “high” represent 1 SD below and above the mean of all countries and average is the mean for all countries. Each component of SES is country-mean centered with standard deviation units above and below the mean on the *x*-axis. Examining differences between the three levels of spending demonstrates a clear pattern that generalizes across all components of SES: The slope for the effect of SES is weaker as the level of social investment increases. It is important to note that this figure especially makes clear the fact that individuals face different levels of depression at the same level of low status depending on the country in which they live. Differences at high levels of SES are not affected, and specifically, depression levels are not worse due to state investments at high levels of status. Put another way, the impact of social policy here is where it is intended to be and does not degrade mental health levels of the advantaged. Generally, this finding suggests that the consequences of fundamental individual differences in social status depend in part on social context.

Finally, we turn to Table 5 to assess our two research questions across disaggregated social investment and protection policies. We present a table indicating whether between- and within-policy differences interactions are significant for the effect of SES.^9^ Each significant interaction is noted by direction (±) and level of significance. In SI policies, we find a consistent pattern of overall between-country SI spending moderating the effects of education, occupation, and income. There are two exceptions: ALMPs do not reduce the positive slope for education squared and between ECEC does not moderate the effect of household occupation. Social protection has much more limited influence on the SES–depression association: Only incapacity spending moderates the effect of household income on depression.

**Table 5.** Disaggregation of Focal Between (B) and Within (W) Interaction Fixed Effects on Within Effects of Socioeconomic Status (*N* = 83,091).

SES Component	Education_w_		Education_w_^2^		Household Occupation_w_		Household Income_w_	
Between and within	B	W	B	W	B	W	B	W
**Social investment**								
ECEC	X+**		X–**				X+***	
Education	X+**		X–**		X+**		X+**	
ALMPs	X+**				X+**		X+*	
Old age care	X+**		X–*		X+*		X+**	
**Social protection**								
Family cash								
Unemployment								
Old age cash								
Health care								
Survivors cash								
Incapacity cash							X+*	
Housing								

*Note*: X marks significant interactions and direction. All within-country effects are denoted by _W_, and all between-country effects are denoted by _B_. Models adjusted for influence include: household income and ECEC (Denmark, Finland, Poland), all components of SES and ALMPs (Denmark for education and household income, Poland and Norway for household occupation), education and old age care (Poland), and household income and incapacity (Denmark and Norway). Data are from Rounds 3, 6, and 7 of the European Social Survey. SES = socioeconomic status; ECEC = early education and care programs; ALMPs = active labor market policies.

* *p* < .05. **p < .01. ***p < .001 (two-tailed).

Out of 24 possible models involving social protection spending, only one has significant interaction effects in comparison to nearly all models of social investment. These findings conform to our expectations that living in countries with higher levels of certain types of spending bestows a mental health advantage for those who live there, specifically through the reduction of status differences in mental health, and that SI spending specifically is more consequential for this association across the SES spectrum.

## Discussion and Conclusion

Our study contributes to the foundational history of research on the association between SES and mental health by incorporating an institutional perspective of social investment and social protection spending and applying it between and within countries cross-nationally across time. This builds on earlier cross-national studies (Niedzwiedz et al. 2016; Präg et al. 2016; Rai et al. 2013) using an analytic strategy that explicitly accounts for between- and within-country trends across time. In doing so, we find a significant within effect of each component of SES on depression at the individual level, adding to evidence consistent with a social causation interpretation of this association (Gangl 2010) outside of the North American context, while also finding significant policy effects that alter this association.

In answering our first research question, we find evidence to support the notion that SI spending is more consequential for the SES–mental health association. Both generally and in terms of specific disaggregated policies, SI reduces mental health inequities between low and high status across all components of SES. Of particular importance is the effect of SI on the education and depression link. We find—in addition to a general mitigation of the implications of status differences—an impact of SI that also linearizes the effect of education and thus potentially extends its impact to both low and high status. This finding contributes to other findings on the nonlinear effects of education in Europe (Bracke et al. 2014). Our findings also are consistent with Niedzwiedz et al.’s (2016) finding of ALMPs reducing educational inequalities in depression, but we generalize this finding to other forms of SI and across components of SES. Social protection has a much more limited impact. The lone significant SP policy that we find is the moderating effect of incapacity cash transfers on the association between household income and depression. Nevertheless, we demonstrate that more generous welfare states matter for reducing mental health inequalities and argue that this article is one of the first to find direct evidence for Thoits’s (2010) conception of cumulative advantage at the macro level where individuals who live in high spending countries enjoy mental health advantages. Our evidence also points to the importance of the timing of policy interventions in the life course because SI policies are typically more focused on formative stages of the life course and less on later life.

Our second research question focusing on within-country change in policies yielded little evidence for the cross-level effects of policy. These findings are in fact consistent with other within-country studies on mental health that observe relatively small changes, both positive and negative, in the level of spending on mental health outcomes in the population (Lee and Wolf 2014; Pak 2020). For example, over the years we observe in this study, spending remains relatively stable across most spending areas in both SI and SP. However, one benefit of our model specification is that we were able to observe cross-national differences in the effect of continuously high versus low levels of spending between countries, which demonstrated that SI policies are important for cross-national differences. Generally, our null findings suggest focusing on longer periods of time with greater spending variation or on specific policies in countries that may be more historically volatile.

Our article indirectly speaks to a core theme in the long literature linking SES to mental health. Going back to at least Faris and Dunham (1939) and including the classic community studies of the mid-twentieth century (Hollingshead and Redlich 1958) and a history of investigations on the causal interpretation of this relationship (Warren 2009; Wheaton 1978), the emphasis throughout has been on consistency of this relationship across time, measures, and samples. This consistency of findings aligns with the central premises of fundamental cause theory (Link and Phelan 1995), with the exception of interest in SES variation by gender and race (Jackson and Williams 2006). However, most of this research is based on North American findings.

Our study suggests that there is variability in the strength of the link between SES and mental health not only at the individual level (Kessler and MacRae 1981) but also at the macro level of state policy while not questioning the consistency of this association overall. Our article adds another layer to the discussion of SES differences in mental health by considering the social context within which SES differences occur. The link between SES and mental health is not exactly immutable or unchanging across social contexts: Clearly, it depends on features of social context not usually specified in the traditional literatures in sociology addressing this issue.

Our results also have broader implications for social policy research in general. By distinguishing the two theoretical pathways of social protection and social investment, we demonstrated that social investment spending more dramatically reduces the inverse link between SES and depression. At the same time, we find no systematic evidence suggesting that changes in the direction or magnitude of spending (i.e., within-country trends) impact the SES–depression association, either due to the general or specific domains of either social investment or social protection policy. In effect, it is the consistent historical presence of spending that matters.

While our study answers important questions about cross-national differences in the link between SES and mental health, it also raises other questions that need to be answered in further research. A central question concerns the mechanisms through which social policies reduce the linkage between SES and depression. Given evidence that more generous welfare states reduce levels of economic strain (Levecque et al. 2011), a plausible next step is to investigate how the role of configurations of stress histories and coping resources at the individual level that play out over the life course—following the tradition of the stress process (Pearlin 1989; Pearlin et al. 1981)—are impacted by social investment and social protection. There are, in fact, some clues in the results in Table 4. Policies that target ECEC and education have broad effects on reducing the mental health disadvantages of low education, occupation, and income, suggesting that the impacts of these policies may operate fundamentally through increased access to educational opportunities, which is consequential for both labor force placement and mobility and indirectly for income. In addition, low status individuals may disproportionately benefit from ECEC and old age care in part because of increased labor force attachment among women (Boeckmann, Misra, and Budig 2015; Costa-Font and Vilaplana-Prieto 2022), in turn indirectly increasing household income while also providing women with mental health benefits (Kessler and McRae 1981). ALMPs may further support this earlier advantage. These policy processes may in turn alter the distribution of chronic stress exposures over lives (Turner et al. 1995) and the development of a sense of control or mastery (Mirowsky and Ross 2007) within countries.

Our study is not without limitations. Due to restrictions caused by the specific waves of the ESS that included the measurement of depression, we have limited waves of data. Ideally, we would want more than the three waves available for analysis and/or more widely spaced waves. For example, it is recommended to have at least four or more waves to detect the complex within-country effects under investigation in this article (Giesselmann and Schmidt-Catran 2019). In addition, our measures do not deal with variation in policy delivery, whether targeted or universal (Brady and Burroway 2012), that may more directly affect depression. Our measures of policy also do not include measures of duration of benefits or their generosity for each type of policy (Scruggs and Allan 2008), which is demonstrated to affect mental well-being (Glass, Simon, and Andersson 2016). Finally, variation in the effect of spending effort during this period itself may have been due to other factors such as the 2008 world financial crisis, an issue that could partially confound observed associations.

Despite these limitations, our analysis contributes theoretically and empirically to the growing body of evidence that suggests social inequality in mental health depends on the policy context one lives within. Our findings also strongly suggest that social policy may be another “fundamental cause” of health inequalities (Beckfield et al. 2015) in addition to previous focuses on individual-level determinants (Link and Phelan 1995). Our findings suggest that the reduction of human suffering is indeed within the control of policymakers, and as such, considerations of policy outcomes should incorporate the understanding that changing policy may also mean changing the balance of suffering across an entire society.

## Supplemental Material

sj-docx-1-hsb-10.1177_00221465231166334 – Supplemental material for The Effect of Welfare State Policy Spending on the Equalization of Socioeconomic Status Disparities in Mental Health Click here for additional data file. Supplemental material, sj-docx-1-hsb-10.1177_00221465231166334 for The Effect of Welfare State Policy Spending on the Equalization of Socioeconomic Status Disparities in Mental Health by Matthew Parbst and Blair Wheaton in Journal of Health and Social Behavior

## Notes

1. Individuals who had more than four items of the index missing were treated as missing at random and deleted.

2. Top coded at the 99.5% of the sample distribution due to extreme values.

3. Because of different codings of household income between the 2006 and 2012 and 2014 waves, 2006 was coded into deciles through collapsing income categories into the appropriate distributional properties. The same procedure is used in Glass et al. (2016).

4. Belgium 2012 was extracted from the Eurostat database. Britain 2012 is derived by averaging across 2011 and 2013 data points.

5. Data extracted in 2017.

6. All data available by request.

7. Original scale was divided by 100.

8. Survey wave is appropriate in this context compared to each survey year because there is no significant differences between years within each wave respectively.

9. Full models are in Appendix D in the online version of the article.
