# Supplementary material for: The Effect of Welfare State Policy Spending on the Equalization of Socioeconomic Status Disparities in Mental Health
Source: J Health Soc Behav. 2023 Apr 25;64(3):336–53. doi: 10.1177/00221465231166334 (PMC10486153; doi:10.1177/00221465231166334)
Supplement: sj-docx-1-hsb-10.1177_00221465231166334 – Supplemental material for The Effect of Welfare State Policy Spending on the Equalization of Socioeconomic Status Disparities in Mental Health [file sj-docx-1-hsb-10.1177_00221465231166334.docx]

**Journal** of **Health**

and **Social Behavior**

OFFICIAL JOURNAL OF THE AMERICAN SOCIOLOGICAL ASSOCIATION

**ONLINE SUPPLEMENT**

**to article in**

Journal of Health and Social Behavior

**The Effect of Welfare State Policy Spending on the Equalization of Socioeconomic Status Disparities in Mental Health**

**Matthew Parbst**

*University of Toronto*

**Blair Wheaton**

*University of Toronto*

## Appendix A: Model Structure

A representative equation for the combined fixed and random effects part of our model is:

$$Depression= \beta_{00}+\beta_{01}\left( {SES}_{w} \right)_{jti}+\beta_{02}\left( {SES}_{b} \right)_{j}+\beta_{03}\left( {Spending}_{w} \right)_{jt}+\beta_{04}\left( {Spending}_{b} \right)_{j}+\beta_{05}\left( {Country Controls}_{w} \right)_{jt}{+ \beta}_{06}\left( {Country Controls}_{b} \right)_{j}+\beta_{07}\left( {Idividual Controls}_{w} \right)_{jti}+\beta_{08}\left( {Idividual Controls}_{b} \right)_{j}+\beta_{09}\left( Survey \right)_{t}+\beta_{10} \left( {{(SES}_{b})}_{j}*{{(Spending}_{w})}_{jt} \right)+\beta_{11} \left( {{(SES}_{w})}_{jti}*{{(Spending}_{b})}_{j} \right)+\beta_{12} \left( {{(SES}_{w})}_{jti}*{{(Spending}_{w})}_{jt} \right)+v_{0j}+v_{1j}\left( {SES}_{w} \right)_{ij}+u_{0jt}+u_{1jt}\left( {SES}_{w} \right)_{ij}+e_{0ijt}$$

The model has two sets of subscripts indicating the within-between coding and the three-level structure of the model where: within effects (*w)*, between effects (*b)*, country-level variables (*j*), country-year variables (*t*), and individuals (*i*) are denoted respectively. Our variable labels include: SES is a component of SES, spending is a stand-in for measures of state spending, survey is a set of dummy variables relating to surveys, individual and country-level controls are noted in groups. Finally, the error portion of our model is distinguished by $v_{0j}$ for country-level random error, $v_{1j}{({SES}_{w})}_{ij}$ for the random coefficient of SES at the country-level, $u_{0jt}$ for error at the country-year level, $u_{1jt}{({SES}_{w})}_{ij}$ for the random coefficient for SES at the country-year level, and $e_{0ijt}$ for the individual error term.

## Appendix B: Cook’s D Assessment Model Overview

| ***Appendix B.1: Cook’s D for all Significant Models for the Moderating Effect Social Investment and Social Protection on Education, Occupation, and Income*** | | | |
| --- | --- | --- | --- |
| ***SES Component*** | Education | Occupation | Income |
| ***Country*** | S.I. | S.I. | S.I. |
| Austria | 0.041 | 0.049 | 0.039 |
| Belgium | 0.090 | 0.093 | 0.166 |
| Czech Republic | 0.185 | 0.148 | 0.132 |
| Denmark | 0.153 | 0.418 | 0.298 |
| Estonia | 0.166 | 0.177 | 0.125 |
| Finland | 0.088 | 0.127 | 0.194 |
| France | 0.079 | 0.108 | 0.108 |
| Germany | 0.080 | 0.119 | 0.079 |
| Ireland | 0.144 | 0.167 | 0.152 |
| Israel | 0.183 | 0.098 | 0.132 |
| Lithuania | 0.265 | 0.304 | 0.299 |
| Netherlands | 0.054 | 0.087 | 0.030 |
| Norway | 0.235 | 0.262 | 0.175 |
| Poland | 0.237 | 0.427 | 0.443 |
| Portugal | 0.400 | 0.675 | 1.473 |
| Sweden | 0.217 | 0.252 | 0.219 |
| Slovenia | 0.142 | 0.123 | 0.111 |
| Slovakia | 0.205 | 0.157 | 0.245 |
| Spain | 0.225 | 0.212 | 0.214 |
| Switzerland | 0.061 | 0.072 | 0.081 |
| United Kingdom | 0.096 | 0.126 | 0.153 |
| Cut-off value (4/N) =.190. Notes: All countries over the Cook’s D cut-off value were removed from analysis to assess their influence on the final model. | | | |

| **Appendix B.2: *Cook’s D for all Significant Models for the Moderating Effect of Disaggregated Social Investment Spending on Education, Occupation, and Income*** | | | | | | | | | | | |
| --- | --- | --- | --- | --- | --- | --- | --- | --- | --- | --- | --- |
| **Policy Area** | Social Investment | | | | | | | | | | |
| ***Program :*** | ECEC | | Education | | | ALMP | | | Old Age Care | | |
| *SES Component:* | Educ. | Inc. | Educ. | Occ. | Inc. | Educ. | Occ. | Inc. | Educ. | Occ. | Inc. |
| Austria | 0.046 | 0.041 | 0.052 | 0.043 | 0.036 | 0.045 | 0.046 | 0.041 | 0.107 | 0.094 | 0.066 |
| Belgium | 0.075 | 0.167 | 0.086 | 0.09 | 0.128 | 0.074 | 0.096 | 0.143 | 0.086 | 0.098 | 0.181 |
| Czech Republic | 0.162 | 0.130 | 0.22 | 0.143 | 0.138 | 0.141 | 0.156 | 0.134 | 0.145 | 0.150 | 0.127 |
| Denmark | 0.131 | 0.111 | 0.173 | 0.105 | 0.068 | 0.107 | 0.121 | 0.111 | 0.127 | 0.149 | 0.102 |
| Estonia | 0.162 | 0.125 | 0.169 | 0.196 | 0.144 | 0.160 | 0.168 | 0.191 | 0.162 | 0.169 | 0.116 |
| Finland | 0.085 | 0.193 | 0.084 | 0.125 | 0.187 | 0.085 | 0.109 | 0.192 | 0.086 | 0.115 | 0.196 |
| France | 0.084 | 0.143 | 0.078 | 0.098 | 0.095 | 0.076 | 0.103 | 0.100 | 0.079 | 0.108 | 0.104 |
| Germany | 0.144 | 0.294 | 0.102 | 0.430 | 0.305 | 0.201 | 0.527 | 0.327 | 0.139 | 0.417 | 0.294 |
| Ireland | 0.147 | 0.166 | 0.147 | 0.165 | 0.157 | 0.152 | 0.191 | 0.252 | 0.147 | 0.164 | 0.157 |
| Israel | 0.179 | 0.155 | 0.161 | 0.096 | 0.131 | 0.185 | 0.087 | 0.187 | 0.184 | 0.090 | 0.144 |
| Lithuania | 0.239 | 0.338 | 0.249 | 0.315 | 0.262 | 0.26 | 0.366 | 0.377 | 0.248 | 0.326 | 0.353 |
| Netherlands | 0.057 | 0.028 | 0.062 | 0.102 | 0.037 | 0.076 | 0.095 | 0.029 | 0.055 | 0.066 | 0.036 |
| Norway | 0.246 | 0.199 | 0.229 | 0.244 | 0.176 | 0.204 | 0.270 | 0.210 | 0.291 | 0.371 | 0.202 |
| Poland | 0.273 | 0.538 | 0.22 | 0.400 | 0.388 | 0.231 | 0.445 | 0.458 | 0.24 | 0.487 | 0.473 |
| Portugal | 0.345 | 1.066 | 0.724 | 0.931 | 2.500 | 0.641 | 0.629 | 0.857 | 0.306 | 0.608 | 1.032 |
| Sweden | 0.166 | 0.192 | 0.193 | 0.227 | 0.193 | 0.163 | 0.226 | 0.186 | 0.168 | 0.243 | 0.221 |
| Slovenia | 0.114 | 0.105 | 0.143 | 0.131 | 0.120 | 0.130 | 0.140 | 0.112 | 0.122 | 0.114 | 0.110 |
| Slovakia | 0.231 | 0.300 | 0.213 | 0.150 | 0.234 | 0.218 | 0.147 | 0.195 | 0.223 | 0.164 | 0.255 |
| Spain | 0.250 | 0.145 | 0.260 | 0.283 | 0.554 | 0.411 | 0.484 | 0.293 | 0.370 | 0.488 | 0.263 |
| Switzerland | 0.060 | 0.092 | 0.141 | 0.110 | 0.232 | 0.057 | 0.106 | 0.097 | 0.057 | 0.100 | 0.101 |
| United Kingdom | 0.095 | 0.154 | 0.095 | 0.129 | 0.150 | 0.104 | 0.127 | 0.158 | 0.095 | 0.144 | 0.161 |
| Cut-off value (4/N) =.190. Notes: All countries over the Cook’s D cut-off value were removed from analysis to assess their influence on the final model. Educ., Occ., and Inc., are abbreviations for education, occupation, and income respectively. | | | | | | | | | | | |

| **Appendix B.3.:  *Cook’s D for all Significant Models for Disaggregated Significant Social Protection Spending Models*** | |
| --- | --- |
| ***Program :*** | Incapacity |
| *SES Component:* | Income |
| Austria | 0.038 |
| Belgium | 0.163 |
| Czech Republic | 0.181 |
| Denmark | 0.060 |
| Estonia | 0.133 |
| Finland | 0.194 |
| France | 0.092 |
| Germany | 0.323 |
| Ireland | 0.180 |
| Israel | 0.131 |
| Lithuania | 0.403 |
| Netherlands | 0.090 |
| Norway | 0.193 |
| Poland | 0.495 |
| Portugal | 1.178 |
| Sweden | 0.259 |
| Slovenia | 0.105 |
| Slovakia | 0.151 |
| Spain | 0.244 |
| Switzerland | 0.073 |
| United Kingdom | 0.161 |
| Cut-off value (4/N) =.190 Notes: All countries over the Cook’s D cut-off value were removed from analysis to assess their influence on the final model. | |

## Appendix C:Within Country Descriptive Information

| **Appendix C.1.: Within Country Descriptives of Social Investment and Social Protection Spending.** | | | | | | | | | | | | |
| --- | --- | --- | --- | --- | --- | --- | --- | --- | --- | --- | --- | --- |
|  | Round 3 | | | Round 6 | | | Round 7 | | | Mean (S.D.) | | |
|  | ***N*** | ***S.I.*** | ***S.P.*** | ***N*** | ***S.I.*** | ***S.P.*** | ***N*** | ***S.I.*** | ***S.P.*** | ***N*** | ***S.I.*** | ***S.P.*** |
| **Country** |  |  |  |  |  |  |  |  |  |  |  |  |
| Austria | 1314 | 6.45 | 23.16 |  |  |  | 1258 | 7.28 | 25.03 | 2572 | 6.86(0.41) | 24.08(0.93) |
| Belgium | 1400 | 7.13 | 22.73 | 1552 | 8.16 | 25.80 | 1480 | 8.42 | 26.62 | 4432 | 7.92(0.55) | 25.10(1.65) |
| Czech Republic |  |  |  | 1248 | 5.00 | 19.70 | 1388 | 5.01 | 18.12 | 2636 | 5.01(0.01) | 18.87(0.78) |
| Denmark | 1277 | 12.31 | 18.22 | 1354 | 12.90 | 20.91 | 1279 | 12.93 | 22.10 | 3910 | 5.94(0.23) | 22.24(0.25) |
| Estonia | 971 | 5.06 | 11.91 | 1845 | 5.41 | 14.79 |  |  |  | 2816 | 5.29(0.17) | 13.80(1.36) |
| Finland | 1661 | 8.35 | 19.50 | 1971 | 9.98 | 22.92 | 1886 | 9.98 | 24.51 | 5518 | 9.48(0.75) | 22.43(2.04) |
| France | 1622 | 7.78 | 25.45 | 1679 | 8.13 | 28.92 | 1640 | 8.16 | 28.97 | 4941 | 8.02(0.17) | 27.79(1.64) |
| Germany | 2021 | 5.59 | 22.64 | 2392 | 6.13 | 22.06 | 2576 | 6.05 | 22.10 | 6989 | 12.72(0.29) | 20.02(1.25) |
| Ireland | 1161 | 5.67 | 14.30 | 1797 | 7.39 | 20.95 | 1741 | 6.32 | 18.33 | 4699 | 6.57(0.70) | 18.34(2.58) |
| Israel |  |  |  | 1528 | 6.95 | 13.36 | 1728 | 7.25 | 13.23 | 3256 | 7.11(0.15) | 13.29(0.67) |
| Lithuania |  |  |  | 1554 | 5.64 | 13.92 | 1652 | 5.52 | 13.85 | 3206 | 5.58(0.06) | 13.88(0.03) |
| Netherlands | 1577 | 7.53 | 13.22 | 1502 | 8.06 | 15.24 | 1658 | 7.88 | 15.27 | 4737 | 7.82(0.23) | 14.58(0.96) |
| Norway | 1598 | 9.41 | 14.91 | 1468 | 10.86 | 16.33 | 1291 | 11.26 | 17.40 | 4357 | 10.45(0.81) | 16.13(1.02) |
| Poland | 1210 | 5.99 | 19.29 | 1360 | 5.72 | 18.22 | 1106 | 5.93 | 18.79 | 3676 | 5.87(0.12) | 18.74(0.45) |
| Portugal | 1093 | 5.84 | 20.84 | 919 | 5.82 | 23.46 | 940 | 5.82 | 22.92 | 2952 | 5.83(0.01) | 22.32(1.15) |
| Slovakia | 922 | 5.03 | 14.29 | 1144 | 4.89 | 16.60 |  |  |  | 2066 | 4.95(0.72) | 15.57(1.15) |
| Slovenia | 987 | 6.39 | 19.87 | 838 | 6.56 | 22.13 | 862 | 5.76 | 21.29 | 2687 | 6.24(0.34) | 21.02(0.94) |
| Spain | 1015 | 5.73 | 19.80 | 1348 | 5.94 | 26.54 | 1346 | 5.90 | 22.47 | 3709 | 5.87(0.08) | 23.22(2.72) |
| Sweden | 1686 | 11.02 | 19.41 | 1600 | 12.68 | 18.42 | 1590 | 12.82 | 18.42 | 4876 | 12.45(0.82) | 18.76(0.47) |
| Switzerland | 1400 | 6.12 | 12.71 | 1173 | 6.15 | 13.30 | 1215 | 6.25 | 13.43 | 3788 | 6.17(0.05) | 13.12(0.32) |
| United Kingdom | 1787 | 6.83 | 16.14 | 1678 | 7.07 | 19.63 | 1803 | 6.76 | 19.53 | 5268 | 6.88(0.13) | 18.41(1.62) |

| ***Appendix C.2.: ESS Round 3 Within Country Descriptives of Depression, Education, Occupation, and Household Income*** | | | | |
| --- | --- | --- | --- | --- |
|  | ***Depression(S.E.)*** | ***Education(S.E.)*** | ***Occupation(S.E.)*** | ***Income (S.E.)*** |
| **Country** |  |  |  |  |
| Austria | 5.67(4.00) | 12.70 (3.09) | 4.67(1.97) | 5.57(1.98) |
| Belgium | 5.32(4.09) | 12.38(3.70) | 4.98(2.21) | 6.08(2.00) |
| Czech Republic | 6.78(4.40) | 13.00(2.43) | 4.39(2.05) | 5.08(2.47) |
| Denmark | 4.69(3.29) | 13.54(4.85) | 5.18(2.24) | 6.90(2.07) |
| Estonia | 7.11(4.16) | 12.31(3.28) | 4.56(2.29) | 2.62(1.28) |
| Finland | 4.95(3.34) | 12.72(4.22) | 4.73(2.28) | 6.02(2.04) |
| France | 5.58(4.25) | 12.74(4.08) | 4.81(2.14) | 5.72(2.06) |
| Germany | 6.14(3.81) | 13.49(3.36) | 4.70(2.09) | 5.62(1.96) |
| Great Britain | 5.73(4.04) | 13.64(3.67) | 4.71(2.15) | 6.27(2.44) |
| Ireland | 4.81(3.74) | 13.01(3.36) | 4.77(2.20) | 6.45(2.33) |
| Israel |  |  |  |  |
| Lithuania |  |  |  |  |
| Netherlands | 5.29(3.77) | 13.41(4.31) | 5.21(2.08) | 5.93(2.09) |
| Norway | 4.19(3.06) | 13.58(3.78) | 4.87(2.11) | 7.62(2.12) |
| Poland | 6.90(4.86) | 11.67(3.34) | 3.94(2.15) | 2.56(1.44) |
| Portugal | 8.01(4.69) | 7.07(4.92) | 3.41(1.95) | 3.75(2.01) |
| Slovakia | 7.51(3.98) | 12.5(3.24) | 4.22(2.15) | 2.91(1.56) |
| Slovenia | 5.61(3.74) | 12.06(3.65) | 4.62(2.17) | 4.14(1.65) |
| Spain | 5.56(4.25) | 12.04(5.49) | 3.95(2.12) | 5.13(2.04) |
| Sweden | 4.93(3.79) | 12.83(3.60) | 4.98(2.14) | 6.42(1.92) |
| Switzerland | 4.74(3.38) | 13.71(3.68) | 5.05(2.11) | 7.59(1.85) |
| United Kingdom | 5.73(4.04) | 13.64(3.67) | 4.71(2.15) | 6.27(2.44) |

| ***Appendix C.3.: ESS Round 6 Within Country Descriptives of Depression, Education, Occupation, and Household Income*** | | | | |
| --- | --- | --- | --- | --- |
|  | ***Depression(S.E.)*** | ***Education(S.E.)*** | ***Occupation(S.E.)*** | ***Income (S.E.)*** |
| **Country** |  |  |  |  |
| Austria |  |  |  |  |
| Belgium | 5.20(3.93) | 13.27(3.70) | 4.96(2.17) | 5.84(2.44) |
| Czech Republic | 7.54(4.79) | 12.95(2.29) | 4.39(2.10) | 5.83(2.51) |
| Denmark | 4.45(3.43) | 13.32(4.83) | 5.00(2.25) | 5.80(2.83) |
| Estonia | 6.81(4.27) | 12.74(3.33) | 4.41(2.30) | 5.83(2.87) |
| Finland | 4.65(3.34) | 13.44(4.06) | 4.64(2.21) | 5.84(2.66) |
| France | 5.64(4.21) | 12.60(3.96) | 4.57 (2.15) | 4.65(2.72) |
| Germany | 5.64(3.74) | 14.09(3.26) | 4.90(2.06) | 5.53(2.74) |
| Great Britain | 5.37(4.03) | 13.49(3.63) | 4.79(2.19) | 5.14(3.02) |
| Ireland | 4.89(4.23) | 14.01(3.59) | 4.31(2.16) | 3.78(2.35) |
| Israel | 6.02 (4.28) | 13.53(3.53) | 5.04(2.21) | 5.08(2.64) |
| Lithuania | 7.49(3.72) | 12.68(3.30) | 3.99(2.26) | 5.74(2.94) |
| Netherlands | 4.87(3.71) | 13.94(4.13) | 4.99(2.14) | 5.97(2.73) |
| Norway | 3.89(3.08) | 13.32(4.37) | 5.25(2.21) | 5.29(2.71) |
| Poland | 6.03(4.85) | 12.44(3.52) | 4.32(2.17) | 5.23(2.69) |
| Portugal | 7.17(7.40) | 4.90(4.75) | 3.17(1.77) | 3.50(1.95) |
| Slovakia | 6.92(3.91) | 12.88(2.86) | 4.09(2.02) | 4.99(2.50) |
| Slovenia | 4.67(3.76) | 12.04(3.57) | 4.52(2.15) | 4.66(2.45) |
| Spain | 6.13(4.45) | 12.78(5.64) | 4.26(2.15) | 4.77(2.91) |
| Sweden | 4.61(3.64) | 13.06(3.40) | 4.96(2.16) | 6.22(2.86) |
| Switzerland | 4.65(3.46) | 11.99(3.73) | 5.30(2.12) | 5.56(2.55) |
| United Kingdom | 5.37(4.03) | 13.49(3.63) | 4.79(2.19) | 5.14(3.02) |

| ***Appendix C.4.: ESS Round 7 Within Country Descriptives of Depression, Education, Occupation, and Household Income*** | | | | |
| --- | --- | --- | --- | --- |
|  | ***Depression(S.E.)*** | ***Education(S.E.)*** | ***Occupation(S.E.)*** | ***Income(S.E.)*** |
| **Country** |  |  |  |  |
| Austria | 4.94(3.71) | 12.45(3.14) | 4.28(2.01) | 4.69(2.43) |
| Belgium | 4.94(3.80) | 13.45(3.68) | 4.95(2.20) | 5.94(2.44) |
| Czech Republic |  |  |  |  |
| Denmark | 4.52(3.65) | 13.54(4.79) | 5.24(2.26) | 5.81(2.88) |
| Estonia |  |  |  |  |
| Finland | 4.33(3.11) | 13.64(4.28) | 4.84(2.29) | 5.66(2.69) |
| France | 5.27(3.89) | 13.05(3.96) | 4.71(2.19) | 5.21(2.82) |
| Germany | 5.44(3.58) | 14.44(3.30) | 5.09(2.06) | 5.93(2.80) |
| Great Britain | 5.31(4.01) | 13.73(3.65) | 4.86(2.18) | 5.04(2.99) |
| Ireland | 4.52(3.88) | 13.98(3.47) | 4.42(2.28) | 4.15(2.51) |
| Israel | 5.70(4.06) | 13.23(3.56) | 4.97(2.33) | 5.16(2.45) |
| Lithuania | 7.41(3.76) | 12.71(3.25) | 3.94(2.22) | 5.26(2.83) |
| Netherlands | 4.54(3.66) | 13.94(3.89) | 5.15(2.10) | 5.91(2.71) |
| Norway | 3.96(3.10) | 14.12(3.71) | 5.38(2.17) | 5.33(2.81) |
| Poland | 5.45(4.69) | 12.5(3.39) | 4.21(2.14) | 5.31(2.78) |
| Portugal | 7.01(4.80) | 9.15(5.25) | 3.94(2.18) | 4.78(2.69) |
| Slovakia | 5.18(3.92) | 12.42 (3.47) | 4.58(2.18) | 4.75(2.58) |
| Slovenia |  |  |  |  |
| Spain | 5.92(4.44) | 12.85(5.53) | 4.19(2.21) | 5.07(2.67) |
| Sweden | 4.76(3.65) | 13.33(3.56) | 5.11(2.16) | 6.36(2.92) |
| Switzerland | 4.10(3.47) | 11.23(3.37) | 5.23(2.15) | 5.40(2.58) |
| United Kingdom | 5.31(4.01) | 13.73(3.65) | 4.86(2.18) | 5.04(2.99) |

## Appendix D: Disaggregated Social Investment and Social Protection Spending

| **Appendix D1.: *Comparing the Moderating Effects of Social Investment Spending on SES and Depression N=83,091*** | | | | | | |
| --- | --- | --- | --- | --- | --- | --- |
| ***Program :*** | | ECEC | | Education | | |
| *SES Component:* | | Educ. | Inc. | Educ. | Occup. | Inc. |
| **Individual** | |  |  |  |  |  |
|  | SES_w_ | -0.785*** | -0.387*** | -1.319*** | -0.483*** | -0.622*** |
|  |  | (0.098) | (0.039) | (0.255) | (0.084) | (0.078) |
|  | Educ^2^_w_ | 0.020*** | 0.008*** | 0.033** | 0.009*** | 0.008*** |
|  |  | (0.003) | (0.001) | (0.008) | (0.001) | (0.001) |
| **Contextual** | |  |  |  |  |  |
|  | Program_w_ | -4.175 | -0.528 | -3.205 | -0.749 | -2.314 |
|  |  | (7.906) | (3.834) | (2.729) | (2.460) | (1.429) |
|  | Program_b_ | -0.420 | -0.009 | -0.392** | -0.301* | -0.375* |
|  |  | (0.436) | (0.653) | (0.115) | (0.112) | (0.122) |
| *Interaction Terms* | |  |  |  |  |  |
| SES_w_*Program_b_ | | 0.512** | 0.142* | 0.163** | 0.044** | 0.061*** |
|  | | (0.124) | (0.050) | (0.046) | (0.015) | (0.014) |
| Educ^2^_b_*Program_b_ | | -0.014** |  | -0.004* |  |  |
|  | | (0.004) |  | (0.001) |  |  |
| SES_w_*Program_w_ | | -0.033 | 0.219* | -0.002 | -0.019 | 0.041 |
|  | | (0.056) | (0.104) | (0.018) | (0.027) | (0.033) |
| P-value <0.05; **P-value <0.01; ***P-value <0.001. All models two-tailed tests. Notes: all within country effects are denoted by _w_  and all between country effects are denoted by _b_ model. Notes: Models adjusted for influence include: household income and ECEC(Denmark, Finland, Poland). Moreover, the within interaction of income and ECEC fails further robustness tests and should be interpreted with caution. | | | | | | |

| **Appendix D2.: *Comparing the Moderating Effects of Social Investment Spending on SES and Depression N=83,091*** | | | | | | | |
| --- | --- | --- | --- | --- | --- | --- | --- |
| ***Program :*** | | ALMP | | | Old Age Care | | |
| *SES Component:* | | Educ. | Occup . | Inc. | Educ. | Occup.. | Inc. |
| **Individual** | |  |  |  |  |  |  |
|  | SES_w_ | -0.661*** | -0.330*** | -0.363*** | -0.564*** | -0.281*** | -0.334*** |
|  |  | (0.103) | (0.027) | (0.034) | (0.066) | (0.021) | (0.021) |
|  | Educ^2^_w_ | 0.016*** | 0.009*** | 0.008*** | 0.014*** | 0.009*** | 0.008*** |
|  |  | (0.003) | (0.001) | (0.001) | (0.002) | (0.001) | (0.001) |
| **Contextual** | |  |  |  |  |  |  |
|  | Program_w_ | 4.114 | -3.359 | -1.640 | 10.939 | 11.897 | 1.671 |
|  |  | (7.822) | (4.754) | (4.592) | (12.891) | (6.34) | (8.011) |
|  | Program_b_ | -0.035 | -0.066 | -0.535 | -0.340 | -0.117 | -0.274 |
|  |  | (0.650) | (0.469) | (0.692) | (0.263) | (0.231) | (0.271) |
| *Interaction Terms* | |  |  |  |  |  |  |
| SES_w_*Program_b_ | | 0.352* | 0.135** | 0.118* | 0.249** | 0.072* | 0.088 |
|  | | (0.136) | (0.036) | (0.044) | (0.079) | (0.025) | (0.025) |
| Educ^2^_b_*Program_b_ | | -0.008 |  |  | -0.006* |  |  |
|  | | (0.004) |  |  | (0.002) |  |  |
| SES_w_*Program_w_ | | -0.045 | -0.167 | -0.131 | -0.106 | -0.234 | 0.013 |
|  | | (0.054) | (0.085) | (0.109) | (0.078) | (0.124) | (0.158) |
| P-value <0.05; **P-value <0.01; ***P-value <0.001. All models two-tailed tests. Notes: all within country effects are denoted by _w_  and all between country effects are denoted by _b_ model. Notes: Models adjusted for influence include: all components of SES and ALMPs (Denmark for education and household income, Poland and Norway for household occupation), education and Old age care (Poland) | | | | | | | |

| **Appendix D.3.: *Disaggregated Social Protection Spending on SES and Depression N=83,091*** | | | |
| --- | --- | --- | --- |
| ***Program :*** | | Incapacity |  |
| *SES Component:* | | Income |  |
| **Individual** | |  |  |
|  | SES_w_ | -0.460*** |  |
|  |  | (0.074) |  |
| **Contextual** | |  |  |
|  | Program_w_ | 0.993 |  |
|  |  | (2.169) |  |
|  | Program_b_ | -1.388* |  |
|  |  | (0.508) |  |
| *Interaction Terms* | |  |  |
| SES_w_ *Program_b_ | | 0.084* |  |
|  | | (0.035) |  |
| SESw *Program_w_ | | -0.059 |  |
|  | | (0.050) |  |
| *P-value <0.05; **P-value <0.01; ***P-value 0.001. Note: all within country effects are denoted by _w_  and all between country effects are denoted by _b._ Notes: Models adjusted for influence include: household income and incapacity (Denmark and Norway). | | |  |
